# Dataset of concentrations of mercury and methylmercury in fish from a tropical river impacted by gold mining in the Colombian Pacific

**DOI:** 10.1016/j.dib.2020.106513

**Published:** 2020-11-06

**Authors:** Carlos Salazar-Camacho, Manuel Salas-Moreno, Roberth Paternina-Uribe, José Marrugo-Negrete, Sergi Díez

**Affiliations:** a Universidad Tecnológica del Chocó, Biology Department - Biosistematic Research Group, Quibdó 270008, Colombia; b Universidad de Córdoba, Carrera 6 No. 76-103, Montería, Córdoba 230003, Colombia; c Department of Environmental Chemistry, Institute of Environmental Assessment and Water Research, IDAEA-CSIC, E-08034 Barcelona, Spain

**Keywords:** Atrato River Basin, THg concentrations, MeHg concentrations, *H. malabaricus*, *C. krausii*, Gold mining, Tropical ecosystems

## Abstract

This data article includes information on the impact of gold mining along five zones of a tropical river in the Pacific region of Colombia. The concentrations of total mercury (THg), total length, mertimercury (MeHg) were determined in 16 species of fish. With this information, it was shown as the concentrations of mercury in fish are influenced by the distribution in the contamination along the Atrato River Basin [1]. Further, THg and MeHg concentrations were related with the trophic level to show biomagnification, and with total length to show bioaccumulation, which is important to establish the potential risk to the environment and also to the health of the inhabitants living along the basin from the consumption of fish.

## Specifications Table

**Table:** 

Subject	Environmental Science
Specific subject area	Pollution, Environmental Chemistry
Type of data	Table
How data were acquired	Wet chemistry for digestions of samples and instrumental analysis for THg and MeHg measurements using a direct Mercury Analyzer, model DMA-80 TRICELL, Milestone Inc., Italy.
Data format	Raw
Parameters for data collection	MeHg percentage, total length, THg and MeHg concentrations, for 842 samples of 16 fish species (3 piscivorous, 4 carnivores, 4 omnivores with tendency to carnivore, 1 omnivore with preference for fish and plant material, 1 omnivore and 3 detritivores)
Description of data collection	Samples fish were collected in zones with the high mining activity [upstream: Rio Quito (RQ), Medio Atrato (MA), Murindó & Vigía del Fuerte (MVF)] to those with the least activity [downstream: Riosucio & Carmen del Darién (RS), and Ciénaga de Unguía & Tumaradó (CUT)] on the Atrato River. Total length was measured, and 20-50 g of the dorsal muscle was cut out for THg and MeHg measurements (UNEP, 1990). The species were identified using Fishbase database (Fishbase, 2020). Kruskal-Wallis, Spearman, Shapiro-Wilks, Kolmogorov-Smirnov, median and standard deviation test were used to make the data analysis [1], which was performed using the R Project Statistical Program version 3.6.1.
Data source location	[Institution: University Technological of Chocó, City/Town/Region: Chocó Country: Colombia Latitude and longitude (and GPS coordinates, if possible) for collected samples/data: Atrato River Basin, the zones were: Rio Quito (5 °, 20’ 53”N, 76 °, 44’ 21” W), Medio Atrato (5 °, 59’ 07”N, 76 °, 46’ 17” W), Murindó (6 °, 58’ 30”N, 76 °, 49’ 37” W) & Vigía del Fuerte (6 °, 56’ 10”N, 76 °, 36’ 10” W), Riosucio (7 °, 26’ 34”N, 76 °, 07’ 12” W) & Carmen del Darién (7 °, 09’ 25”N, 76 °, 57’ 58” W), and Ciénaga de Unguía (8 °, 01’ 23”N, 77 °, 02’ 31” W) & Tumaradó (7 °, 46’ 07”N, 77 °, 02’ 21” W)
Data accessibility	With the article
Related research article	C. Salazar-Camacho, M. Salas-Moreno, R. Paternina-Uribe, J. Marrugo-Negrete, S. Díez, Mercury species in fish from a tropical river highly impacted by gold mining at the Colombian Pacific region, Chemosphere. https://doi.org/10.1016/j.chemosphere.2020.128478

## Value of the Data

• The data set includes valuable MeHg concentrations in fish samples (*n* = 520) collected in the Atrato River Basin, a tropical ecosystem highly impacted by goldmining.
• The data set contains extremely scarce and therefore very valuable MeHg concentration values in fish, which are more suitable than THg values for human health risk assessment.
• The data set describes the distribution of the contamination by mercury in five areas of the Atrato River basin. Also show the correlations between THg (n = 842) and MeHg concentrations and the total length of the fish per area.
• The data set can be used as a reference to identify the most polluted areas throughout the basin, establish possible environmental effects and the risk to human health from fish consumption.

## 1 Data Description

In this article, we will describe data on mercury concentrations in fish samples of 16 different species, from five areas of the Atrato River Basin, a tropical ecosystem highly impacted by gold mining [1]. The data set was summarized in Table 1, Table 2, Table 3, Table 4, Table 5. They describe the THg and MeHg concentrations and length of fish caught in the areas of: Rio Quito (RQ), Medio Atrato (MA), Murindó & Vigía del Fuerte (MVF), Riosucio & Carmen del Darién (RS) and Ciénaga Unguía & Tumaradó (CUT), respectively. For samples of fish with carnivore habits, the tables shown that the WHO limit for the protection of populations at risk (200 µg/kg^−1^ w/w) [3] is exceeded in 27.1% (*n* = 42) for RQ, 37.7% (*n* = 75) for MA, 53.1% (*n* = 86) for MVF, 37.7% (*n* = 92) for RS and 8.5% (*n* = 7) for CUT zones. Within of this samples, 4.5% (*n* = 7) for RQ, 11.6% (*n* = 23) for MA, 37.7% (*n* = 61) for MVF, 11.5% (*n* = 28) for RS and 2.4% (*n* = 2) for CUT, exceeded the WHO maximum limit of THg in fish for human consumption (500 µg/kg^−1^ w/w) [2]. For samples of fish with non-carnivore habits, the WHO limit of 200 µg/kg^−1^ w/w) [3] was exceeded in 4.5% (*n* = 7) for RQ, and 1.2% (*n* = 1) for CUT. Within of this samples, 2.6% (*n* = 4) for RQ exceeded the WHO limit 500 µg/kg^−1^ w/w) [2]. The data also show that methylmercury is the mercury species found in highest queantity among fish samples analyzed (68.1% - 98.8%); therefore, the risk of mercury contamination from fish ingestion, especially in the inhabitants of the MVF zone, is high.

**Table 1:** Total mercury concentrations (THg), total length, methylmercury concentrations (MeHg) and percentage of methylmercury in fish captured in the Rio Quito (RQ) zone, Atrato River Basin, Colombia. P: piscivore, C: carnivore, OC: omnivore with a tendency to carnivore, O: omnivore, D: detritivore. The RQ site is located in the south-central zone of the Chocó Department, and the fish samples were caught in the Quito River within 5 °25′25′′ N 76 °43′29′′ W and 5 °41′07′′ N 76 °39′58′′ W.

Species	THg (µg kg^−1^)	Total length (cm)	MeHg (µg kg^−1^)	% MeHg
***Hoplias malabaricus (P)***	185.20	28.3	172.23	93.0
	376.39	30.0	358.04	95.1
	299.62	25.7	272.65	91.0
	156.93	22.6	136.94	87.3
	58.02	20.3	53.96	93.0
	411.92	26.5	388.09	94.2
	625.39	33.7	598.61	95.7
	908.41	34.5	874.82	96.3
	522.47	30.4	475.89	91.1
	410.04	24.8	382.34	93.2
	375.55	28.1	329.26	87.7
	56.50	18.8	48.55	85.9
	87.43	19.3	73.31	83.8
	260.88	23.7		
	130.10	21.2		
	134.91	24.6		
	286.64	24.1		
	1,254.59	36.9		
	408.23	23.3		
***Ctenolucius beani (C)***	1,191.06	33.0	1,087.00	91.3
	422.63	38.8	381.22	90.2
	129.72	20.3	120.10	92.6
	335.61	29.1	316.83	94.4
	265.50	24.7		
	123.53	19.6		
***Caquetaia umbrifera (C)***	176.82	19.0	154.94	87.6
	165.70	18.5	142.37	85.9
***Sternopygus macrurus (C)***	250.43	42.2	221.66	88.5
	153.43	32.5	137.43	89.6
	42.70	28.4	35.78	83.8
	79.21	25.2	67.14	84.8
	83.12	27.0		
***Caquetaia kraussi (OC)***	605.34	27.8	582.97	96.3
	323.12	23.2	305.50	94.5
	384.56	25.8	347.64	90.4
	202.00	19.8	186.86	92.5
	272.44	19.0	242.37	89.0
	397.00	21.7	359.21	90.5
	80.85	18.0	65.19	80.6
	118.20	18.5		
	158.11	16.0		
	68.20	17.4		
	74.04	16.0		
	266.00	18.8		
***Rhamdia quelen (OC)***	714.00	24.2	660.46	92.5
	404.05	23.5	377.62	93.5
	385.55	22.6	347.87	90.2
	445.92	23.8	405.79	91.0
	280.81	21.6	254.23	90.5
	292.92	24.0	257.90	88.1
	409.48	20.3	362.51	88.5
	228.07	24.5	207.54	91.0
	356.30	22.0	320.23	89.9
	63.86	28.0	56.76	88.9
	263.58	20.2	203.14	77.1
	273.96	20.9	231.59	84.5
	30.55	16.5	22.80	74.6
	50.69	17.1	42.31	83.5
	51.74	22.0	39.76	76.9
	313.32	19.2		
	211.45	23.2		
	96.17	20.7		
	146.34	20.8		
	115.60	18.2		
	95.16	22.1		
***Astyanax fasciatus (OC)***	378.74	16.2	344.65	91.0
	322.31	14.5	298.43	92.6
	293.06	16.3	256.78	87.6
	273.36	17.1	244.88	89.6
	161.54	15.4	150.77	93.3
	143.46	14.8	123.23	85.9
	140.00	10.8	130.46	93.2
	139.17	10.7	128.00	92.0
	136.43	12.6	126.54	92.8
	123.56	10.7	105.66	85.5
	121.72	11.2	107.22	88.1
	118.60	8.5	104.34	88.0
	91.06	12.5	66.29	72.8
	83.26	14.0	62.96	75.6
	89.43	13.0	81.32	90.9
	65.20	14.0	60.41	92.6
	98.71	10.8	90.95	92.1
	118.38	8.46		
	117.19	11.3		
	114.75	9.1		
	108.33	12.4		
	101.36	6.4		
	101.31	8.2		
	101.05	10.5		
	97.76	6.9		
	91.64	7.6		
	90.98	6.3		
***Pimelodus punctatus (OC)***	145.60	25.4	122.44	84.1
	136.30	22.8	112.77	82.7
	277.40	24.8	243.63	87.8
	125.50	17.5		
	76.80	23.1		
***Leporinus muyscorum (O)***	65.70	30.2	61.23	93.2
	34.70	33.5	32.15	92.6
	221.50	44.2	210.76	95.2
	183.80	28.7	171.85	93.5
	143.10	32.6	132.75	92.8
	103.42	31.0	88.61	85.7
	53.40	29.1		
	32.50	24.6		
***Prochilodus magdalenae (D)***	875.07	38.7	839.93	96.0
	635.70	35.4	614.95	96.7
	533.20	36.3	517.92	97.1
	576.30	35.8	554.63	96.2
	108.40	36.3	103.34	95.3
	459.46	37.2	429.68	93.5
	322.79	37.1	307.92	95.4
	92.66	29.6	87.49	94.4
	39.92	18.4	36.28	90.9
	70.91	29.3		
	57.84	24.6		
	35.63	16.7		
	74.39	27.3		
	116.36	28.5		
	112.04	29.1		
	49.40	23.8		
***Hypostomus hondae (D)***	69.29	15.7	56.89	82.1
	49.13	14.5	37.80	76.9
	30.49	16.7	25.12	82.4
	27.35	18.6	22.51	82.3
	24.88	15.8	20.74	83.4
	83.61	26.7	64.77	77.5
	134.14	25.3	104.52	77.9
	125.49	21.8	102.46	81.6
	154.30	27.1	111.80	72.5
	44.61	25.8	33.90	76.0
	49.78	20.5	38.29	76.9
	49.10	11.2	42.39	86.3
	43.00	12.2	33.61	78.2
	43.94	11.4	30.98	70.5
	55.84	11.3	39.96	71.6
	45.21	11.2	33.58	74.3
	25.67	16.0	18.83	73.4
	15.95	14.0	12.78	80.1
	19.28	14.2	13.52	70.1
	19.63	12.0	13.65	69.5
	61.13	9.5		
	56.14	10.9		
	16.94	14.1		
	31.05	14.6		
	120.33	18.5		
	120.67	20.4		
	110.84	18.7		
	114.56	25.3		
***Cyphocharax magdalenae (D)***	26.58	10.2	22.40	84.3
	31.35	17.5	25.80	82.3
	16.38	9.5	12.64	77.2
	26.23	12.6	20.90	79.7
	30.50	15.0		
	32.57	14.7

**Table 2:** Total mercury concentrations (THg). total length. methylmercury concentrations (MeHg) and percentage of methylmercury in fish captured in the Medio Atrato (MA) zone, Atrato River Basin. Colombia. P: piscivore, C: carnivore, OC: omnivore with a tendency to carnivore, OPV: omnivore with preference for fish and vegetal material, O: omnivore, D: detritivore. The MA site is located in the south-central zone of the Chocó Department (north of Río Quito zone), and the fish samples were caught in the Atrato River within 5 °52′01′′ N 76 °42′33′′ W and 6 °10′13′′ N 76 °42′56′′ W.

Species	THg (µg kg^−1^)	Total length (cm)	MeHg (µg kg^−1^)	% MeHg
***Ageneiosus pardalis (P)***	1,318.72	53.2	1,254.34	95.1
	1,614.15	58.4	1,576.21	97.7
	958.52	36.3	892.43	93.1
	1,403.13	63.5	1,358.73	96.8
	316.85	42.6	288.54	91.1
	150.67	30.4		
	132.76	18.7		
	142.76	20.7		
***Hoplias malabaricus (P)***	1,334.50	37.6	1,289.74	96.6
	1,304.50	36.2	1,218.74	93.4
	1,065.40	32.8	998.75	93.7
	708.47	34.6	686.79	96.9
	546.01	27.5	539.56	98.8
	529.70	30.6	509.55	96.2
	528.31	28.2	511.64	96.8
	516.33	33.4	502.75	97.4
	468.54	32.5	428.76	91.5
	452.54	25.7	432.99	95.7
	415.44	26.7	387.46	93.3
	374.16	27.8		
	344.16	25.2		
	313.42	25.0		
	333.42	32.2		
	263.03	23.8		
	181.53	24.5		
***Trachelyopterus fisheri (P)***	518.32	26.6	487.45	94.0
	441.35	25.2	401.60	91.0
	348.19	24.1	317.87	91.3
	412.38	21.5	395.16	95.8
	289.51	20.2		
***Pseudopimelodus schultzi (C)***	3,112.62	57.2	3,034.75	97.5
	2,977.69	57.0	2,775.76	93.2
	2,206.35	46.8	2,165.77	98.2
	2,035.61	46.3	1,967.87	96.7
	486.63	63.9	467.89	96.1
	472.18	47.5	448.98	95.1
	442.02	63.1	420.56	95.1
	441.49	47.5	423.65	96.0
	309.85	53.3		
	304.06	49.8		
	263.90	44.3		
	363.12	50.5		
	293.21	43.2		
***Ctenolucius beani (C)***	461.34	32.6	438.56	95.1
	423.87	27.4	387.40	91.4
	276.21	22.1	260.72	94.4
	213.50	26.5	187.43	87.8
	373.50	29.7		
***Sternopygus macrurus (C)***	1,611.32	58.4	1,496.73	92.9
	978.65	52.4	892.54	91.2
	177.55	34.3	156.49	88.1
	138.30	32.7	118.39	85.6
	67.25	25.1		
	158.93	30.8		
***Caquetaia kraussii (OC)***	655.64	28.1	602.46	91.9
	297.16	28.0	276.46	93.0
	284.05	27.5	274.84	96.8
	253.99	26.5	234.21	92.2
	185.73	27.3	173.46	93.4
	181.50	27.7	165.78	91.3
	181.44	24.7	178.47	98.4
	177.06	25.3	156.45	88.4
	176.63	25.1	167.90	95.1
	158.13	26.0	147.47	93.3
	136.60	22.4	128.99	94.4
	110.22	23.3	101.32	91.9
	109.44	23.0	99.46	90.9
	85.85	21.5	73.57	85.7
	83.77	21.7		
	83.02	20.2		
	70.65	18.2		
	48.24	16.0		
	71.26	19.4		
	83.97	19.0		
	81.96	17.5		
***Rhamdia quelen (OC)***	1,160.98	25.2	1,098.10	94.6
	409.82	24.5	398.54	97.2
	363.50	23.6	352.53	97.0
	235.01	22.8	216.94	92.3
	227.00	23.3	216.72	95.5
	217.98	24.7	208.78	95.8
	196.70	20.3	183.82	93.5
	168.46	24.5	143.46	85.2
	167.25	22.1	158.63	94.8
	166.65	19.8	153.55	92.1
	165.76	24.2	150.45	90.8
	145.32	20.7		
	132.59	21.0		
	124.57	18.3		
	114.60	23.4		
	111.76	18.1		
	111.02	21.6		
	91.70	17.4		
	66.55	19.1		
	60.11	17.6		
	45.27	17.0		
***Astyanax fasciatus (OC)***	636.59	18.2	588.66	92.5
	517.80	15.5	489.32	94.5
	470.15	17.3	456.25	97.0
	368.74	15.1	354.65	96.2
	312.31	16.4	298.43	95.6
	283.06	15.8	256.78	90.7
	273.36	12.8	234.88	85.9
	272.02	11.7	254.23	93.5
	261.79	14.6	235.67	90.0
	244.88	14.7	234.23	95.7
	231.50	12.2	216.47	93.5
	223.46	9.5	213.55	95.6
	222.03	11.5	212.99	95.9
	219.13	12.3	188.54	86.0
	217.53	10.1	195.36	89.8
	212.82	13.4	186.47	87.6
	201.60	7.4	189.43	94.0
	197.27	9.2	188.68	95.6
	184.81	11.5		
	170.75	7.9		
	166.90	10.6		
	101.36	8.3		
	101.31	6.7		
	101.05	6.4		
	97.76	8.4		
	91.64	6.3		
	90.98	8.2		
	89.08	9.2		
	88.38	7.7		
	83.35	7.0		
	81.48	6.3		
***Andinoacara pulcher (OPV)***	288.80	15.8	257.65	89.2
	211.65	12.3	195.21	92.2
	192.81	15.1	178.33	92.5
	191.87	10.2	166.47	86.8
	190.56	11.8	168.99	88.7
	187.97	14.2	164.98	87.8
	168.95	12.0	149.68	88.6
	168.13	11.3	154.56	91.9
	166.55	13.7	145.68	87.5
	57.52	9.8	49.32	85.7
	32.58	7.8	27.82	85.4
	148.43	10.5	123.66	83.3
	144.70	13.6	117.79	81.4
	142.26	13.8		
	97.50	10.2		
	85.09	9.6		
	134.13	14.5		
	129.54	12.1		
	127.68	10.5		
	122.65	10.1		
	116.09	8.4		
	112.37	8.7		
	106.18	9.4		
***Leporinus muyscorum (O)***	139.98	28.0	130.45	93.2
	139.12	29.0	128.88	92.6
	122.71	29.0	116.76	95.2
	96.11	32.0	89.86	93.5
	52.80	35.0	48.98	92.8
	112.40	31.0	96.30	85.7
	143.40	33.2	126.80	88.4
	70.20	30.6		
	80.70	28.5		
	133.40	34.0		
***Prochilodus magdalenae (D)***	142.59	31.0	136.70	95.9
	120.46	38.0	115.77	96.1
	102.25	35.0	99.32	97.1
	101.57	36.0	97.75	96.2
	100.43	34.0	97.54	97.1
	93.96	38.0	87.87	93.5
	88.15	33.0	84.09	95.4
	87.22	32.0	75.77	86.9
	83.77	34.0	80.34	95.9
	75.57	29.0		
	70.17	29.0		
	68.54	24.0		
	52.58	24.0		
	47.53	38.0		
	45.71	33.0		
***Hypostomus hondae (D)***	241.09	28.7	201.46	83.6
	220.73	36.9	184.58	83.6
	200.72	30.8	165.34	82.4
	196.70	26.8	161.93	82.3
	176.14	25.6	146.86	83.4
	172.85	19.0	154.78	89.5
	169.91	18.2	129.85	76.4
	156.34	22.0	141.24	90.3
	144.80	29.1	106.80	73.8
	137.86	20.9	106.42	77.2
	135.15	17.7	111.21	82.3
	132.92	24.3	101.93	76.7
	132.77	17.2	101.47	76.4
	126.33	17.0	95.75	75.8
	124.67	18.2		
	120.84	20.1		
	117.56	18.5		
	116.77	25.5		
	112.35	23.3		
	95.58	22.5		
	94.41	18.4		
	88.27	21.6		
	79.05	19.5		
	77.64	18.3

**Table 3:** Total mercury concentrations (THg), total length, methylmercury concentrations (MeHg) and percentage of methylmercury in fish captured in the Murindó & Vigía del Fuerte (MVF) zone, Atrato River Basin, Colombia. P: piscivore, C: carnivore, OC: omnivore with a tendency to carnivore, OPV: omnivore with preference for fish and vegetal material, O: omnivore, D: detritivore. The MVF site is located in the western zone of the Antioquia Department (east of the Chocó Department), and the fish samples were caught in the Atrato River within 6 °34′59′′ N 76 °53′57′′ W and 6 °58′38′′ N 76 °49′23′′ W.

Species	THg (µg kg^−1^)	Total length (cm)	MeHg (µg kg^−1^)	% MeHg
***Ageneiosus pardalis (P)***	1,440.74	66.3	1,412.77	98.1
	1,301.65	58.4	1,281.30	98.4
	1,033.01	55.0	955.33	92.5
	708.00	28.0	674.32	95.2
	693.78	30.0	645.90	93.1
	684.01	27.0	666.43	97.4
	654.19	29.0	623.41	95.3
	842.91	26.0	800.43	95.0
	574.39	27.0	554.76	96.6
	926.73	25.5	900.20	97.1
	681.67	26.3	643.44	94.4
	779.46	25.0	744.88	95.6
	809.30	25.8	776.30	95.9
	867.41	37.0	822.70	94.8
	815.48	26.1	767.89	94.2
	562.90	26.0	529.12	94.0
	808.85	29.7	766.11	94.7
	435.86	20.9		
	156.90	25.0		
	594.01	27.1		
	829.77	33.0		
	979.51	53.6		
	950.12	45.3		
	264.60	22.0		
	754.73	28.0		
	936.60	37.5		
	625.65	27.5		
	494.47	27.0		
**Hoplias malabaricus (P)**	798.79	36.0	779.98	97.6
	657.62	34.0	614.39	93.4
	721.04	38.0	675.94	93.7
	600.78	33.0	582.40	96.9
	753.89	35.0	740.46	98.2
	542.35	37.0	521.73	96.2
	575.42	39.0	557.26	96.8
	727.49	36.0	708.36	97.4
	577.88	38.0	551.93	95.5
	732.58	32.0	700.94	95.7
	726.14	34.5		
	614.98	33.6		
	797.29	39.0		
	524.57	28.8		
	590.04	32.0		
	209.99	27.3		
***Trachelyopterus fisheri (P)***	418.82	17.2	398.06	95.0
	731.44	24.0	680.19	93.0
	387.38	21.0	342.03	88.3
	1032.54	27.5	1010.07	97.8
	842.86	24.2	794.90	94.3
	808.40	25.7	782.53	96.8
	692.04	23.3		
	748.74	25.0		
	241.80	18.1		
*Ctenolucius beani (C)*	961.52	30.0	923.65	96.1
	584.73	33.4	546.11	93.4
	506.25	29.0	480.90	95.0
	415.42	24.7	368.85	88.8
	414.67	20.6		
*Sternopygus macrurus (C)*	657.14	67.1	610.23	92.9
	1,345.98	70.5	1,308.14	97.2
*Caquetaia kraussii (OC)*	293.33	23.0	275.40	93.9
	586.86	23.0	551.83	94.0
	429.84	20.5	420.20	97.8
	143.80	20.0	132.60	92.2
	761.87	22.0	711.52	93.4
	552.48	20.0	504.63	91.3
	880.19	21.0	865.78	98.4
	247.83	17.0	228.90	92.4
	761.19	19.5	731.19	96.1
	461.21	30.0	439.33	95.3
	514.01	26.2		
	387.24	20.0		
	417.81	20.3		
	424.98	20.3		
	482.04	21.0		
	382.99	19.6		
*Rhamdia quelen (OC)*	519.84	27.1	496.88	95.6
	456.27	27.5	446.45	97.8
	905.14	29.0	882.35	97.5
	297.34	27.0	277.45	93.3
	640.22	28.0	617.64	96.5
	472.00	26.0	456.80	96.8
	529.02	27.0	503.91	95.3
	668.28	28.8		
	376.46	26.0		
	400.55	26.0		
	91.84	17.1		
*Astyanax fasciatus (OC)*	153.27	8.5	143.26	93.5
	320.60	10.0	309.38	96.5
	84.78	8.0	78.04	92.0
	46.96	6.7	41.88	89.2
	54.89	6.2	51.35	93.6
	58.73	7.5		
	290.84	12.2		
*Andinoacara pulcher (OPV)*	29.85	7.0	24.60	82.4
	35.94	8.7	31.35	87.2
*Leporinus muyscorum (O)*	32.69	27.0	27.70	84.7
	30.17	25.0	25.10	83.2
	96.64	27.5	84.40	87.3
	70.69	24.1	65.70	92.9
*Prochilodus magdalenae (D)*	94.27	24.0	86.07	91.3
	163.32	23.0	147.28	90.2
	192.47	24.0	171.70	89.2
	85.00	24.0	71.52	84.1
	172.90	24.5	160.97	93.1
	149.34	23.0	132.02	88.4
	236.15	23.0	223.63	94.7
	194.18	24.0	185.06	95.3
	114.72	24.0	101.07	88.1
	151.42	23.0	126.59	83.6
	61.05	28.0	53.30	87.3
	85.27	24.0	75.81	88.9
	111.70	24.5	101.76	91.1
	56.20	25.0	48.64	86.5
	93.09	23.2	84.34	90.6
	63.63	21.6	52.97	83.2
	50.51	18.8	45.61	90.3
	87.53	22.0	81.58	93.2
	91.11	26.0	84.73	93.0
	101.73	24.2	90.80	89.3
	70.70	28.8	61.88	87.5
	111.43	32.0	102.96	92.4
	138.45	30.0	130.36	94.2
	120.72	25.0	115.65	95.8
	32.03	18.5	27.80	86.8
	190.18	27.0	179.40	94.3
	125.64	28.0	119.56	95.2
	114.11	26.0		
	159.30	23.0		
	144.11	31.0		
	35.81	23.4		
	24.26	19.4		
	217.16	29.4		
	148.47	23.4		
	108.52	28.0		
	120.07	25.0		
	129.90	25.2		
	93.32	26.5		
	89.25	29.0		
	141.70	32.0		
	112.31	30.0		
	100.19	23.0		
	135.57	26.0		
	119.47	24.0		
	107.17	25.2		
	119.01	22.0		
	111.10	31.0		
	115.02	30.0		
*Hypostomus hondae (D)*	43.51	23.5	37.23	85.6
	14.08	22.7	12.20	86.6
	29.45	22.0	25.73	87.4
	65.05	25.5	56.19	86.4
	18.81	16.0	15.53	82.5
	30.36	17.0	26.24	86.4
	44.84	25.4	40.51	90.3
	15.60	16.1		
	58.11	25.0		
	11.04	15.0		
	43.10	24.0		
	70.88	26.0		
	106.55	35.4		
	11.57	14.2

**Table 4:** Total mercury concentrations (THg), total length, methylmercury concentrations (MeHg) and percentage of methylmercury in fish captured in the Riosucio & Carmen del Darién (RS) zone, Atrato River Basin, Colombia. P: piscivore, C: carnivore, OC: omnivore with a tendency to carnivore, OPV: omnivore with preference for fish and vegetal material, O: omnivore, D: detritivore. The RS site is located in the northern zone of the Chocó Department, and the fish samples were caught in the Atrato River within 7 °09′25′′ N 76 °58′04′′ W and 7 °26′47′′ N 77 °07′04′′ W.

Species	THg (µg kg^−1^)	Total length (cm)	MeHg (µg kg^−1^)	% MeHg
***Ageneiosus pardalis (P)***	690.45	36.5	661.45	95.8
	495.92	47.3	467.90	94.3
	341.86	35.4	312.76	91.5
	996.30	40.7	943.12	94.7
	563.04	47.4	543.81	96.6
	502.21	40.5	467.70	93.1
	311.92	40.1	281.34	90.2
	1,102.15	50.8	1,078.43	97.8
	462.14	45.0	413.88	89.6
	664.11	47.6	621.74	93.6
	682.62	67.0	632.55	92.7
	724.77	29.1		
	715.32	33.0		
	747.78	28.8		
	446.28	33.5		
	449.69	31.0		
	513.32	30.0		
	318.94	33.0		
***Hoplias malabaricus (P)***	262.50	36.7	247.13	94.1
	170.30	30.2	152.38	89.5
	214.30	30.1	189.30	88.3
	416.60	36.0	397.44	95.4
	305.10	30.5	288.74	94.6
	401.40	37.0	373.30	93.0
	328.10	32.5	292.13	89.0
	436.80	38.9	411.22	94.1
	426.50	40.2	390.65	91.6
	123.30	22.1	104.67	84.9
	456.90	33.5	434.92	95.2
	180.30	25.7		
	178.40	24.5		
	135.20	22.8		
	300.50	28.5		
	157.80	23.5		
	225.40	27.5		
	286.60	26.7		
	343.30	31.2		
	282.50	33.0		
	366.40	40.0		
	352.96	38.0		
	474.79	37.6		
	300.19	40.0		
	578.00	36.0		
	571.01	32.0		
	819.33	36.0		
	1,040.13	40.0		
***Trachelyopterus fisheri (P)***	345.34	20.6	321.75	93.2
	374.27	25.8	334.65	89.4
	314.64	22.1	292.16	92.9
	270.19	20.1	235.47	87.1
	243.43	19.7		
	213.43	19.2		
***Pseudopimelodus schultzi (P)***	567.16	52.3	552.98	97.5
	453.29	58.6	422.55	93.2
	484.34	54.8	436.53	90.1
	423.83	48.7	409.73	96.7
	297.46	55.6	276.00	92.8
	335.67	43.7		
	223.24	48.8		
	291.89	47.5		
	412.90	47.7		
***Ctenolucius beani (P)***	165.32	28.1	150.21	90.9
	231.31	25.9	217.87	94.2
	192.62	26.8	178.30	92.6
	181.76	23.5	160.51	88.3
	108.32	18.4		
	137.32	22.4		
	844.22	30.0		
	1,005.70	29.0		
***Caquetaia umbrifera (P)***	125.65	22.7	105.40	83.9
	238.72	27.8	219.45	91.9
	201.18	25.8	187.70	93.3
	175.30	24.3	154.12	87.9
	218.54	26.1	188.33	86.2
	729.34	23.5	689.25	94.5
	256.60	24.0	220.43	85.9
	542.34	25.0	500.18	92.2
	731.63	27.5	702.76	96.1
	112.50	19.5		
	449.53	23.0		
	515.50	24.5		
	551.87	25.0		
***Sternopygus macrurus (P)***	438.65	70.0	402.45	91.7
	274.03	49.0	237.67	86.7
	234.89	30.0	200.08	85.2
***Caquetaia kraussii (OC)***	218.00	26.2	200.56	92.0
	247.74	27.4	218.72	88.3
	237.16	21.0	208.19	87.8
	184.58	21.5	164.82	89.3
	126.49	23.0	109.93	86.9
	117.41	20.7	98.02	83.5
	300.13	25.2	286.12	95.3
	265.95	24.0	254.67	95.8
	46.65	18.3	40.92	87.7
	372.33	27.6	359.55	96.6
	46.55	18.2		
	44.20	19.3		
	36.91	17.7		
	143.13	22.6		
	171.21	23.1		
	290.42	25.8		
	309.15	26.5		
	106.99	21.0		
	201.31	21.5		
	537.40	22.8		
	337.98	21.5		
	293.58	24.0		
	113.94	20.0		
	314.35	25.0		
	292.59	23.0		
	745.26	21.5		
	308.04	26.0		
	640.87	20.0		
	622.16	23.0		
	661.70	21.0		
***Rhamdia quelen (OC)***	112.30	21.4	104.19	92.8
	135.50	23.1	127.31	94.0
	155.10	23.0	139.14	89.7
	148.80	22.5	135.41	91.0
	127.00	22.8	114.57	90.2
	88.09	20.7	78.16	88.7
	81.40	20.1	70.08	86.1
	98.32	21.0	89.48	91.0
	158.30	23.2	140.05	88.5
	89.50	19.3		
	123.70	23.0		
	90.60	21.3		
	82.60	20.5		
	78.70	18.7		
	34.77	17.0		
	25.69	17.5		
***Astyanax fasciatus (OC)***	52.16	6.2	48.47	92.9
	182.30	7.7	168.90	92.6
	201.03	9.5	182.94	91.0
	211.99	16.2	198.91	93.8
	39.63	7.2	36.65	92.5
	38.16	6.5	34.98	91.7
	33.07	6.2	30.58	92.5
	32.05	6.0	28.52	89.0
	158.10	13.8	150.60	95.3
	104.39	10.5	88.50	84.8
	102.40	7.7		
	56.04	8.1		
	49.10	6.6		
	42.04	6.0		
	30.26	6.0		
	26.89	6.6		
	74.60	7.2		
***Pimelodus punctatus (OC)***	127.80	23.7	108.21	84.7
	45.30	17.0	35.40	78.1
	52.60	15.0	40.65	77.3
	91.20	18.9	73.87	81.0
	111.40	20.5		
	24.40	16.5		
***Andinoacara pulcher (OPV)***	141.69	13.6	121.68	85.9
	134.99	14.1	118.56	87.8
	105.72	9.6	96.77	91.5
	99.79	8.8	87.68	87.9
	80.95	11.3	67.55	83.4
	60.31	10.1	56.33	93.4
	164.53	14.8	154.44	93.9
	154.23	10.4	126.46	82.0
	112.16	9.5	88.60	79.0
	56.80	10.2	47.45	83.5
	34.50	7.3		
	24.10	7.6		
	77.22	8.9		
	68.50	10.6		
	56.40	9.1		
	21.30	8.8		
***Leporinus muyscorum (O)***	77.50	36.5	70.20	90.6
	67.23	35.0	60.70	90.3
	29.90	37.0	25.10	83.9
	221.89	52.0	207.50	93.5
	167.46	30.0	150.20	89.7
	88.74	23.0	81.70	92.1
	227.80	43.6	198.43	87.1
	150.90	33.7	129.54	85.8
	190.10	38.3	163.22	85.9
	98.93	31.0	81.85	82.7
	185.45	35.0		
	199.89	30.0		
	226.19	35.0		
	301.78	33.0		
	89.35	35.0		
	176.00	32.5		
	123.45	32.0		
***Prochilodus magdalenae (D)***	118.02	24.0	114.20	96.8
	26.91	27.0	20.20	75.1
	58.55	28.0	47.40	81.0
	52.69	28.0	46.10	87.5
	25.85	28.0	17.60	68.1
	57.25	30.0	50.70	88.6
	84.09	23.0	77.14	91.7
	41.51	27.0	32.80	79.0
	77.05	29.0	64.90	84.2
	98.23	20.0	92.40	94.1
	59.39	33.0	52.70	88.7
	112.43	30.0	100.90	89.7
	80.43	34.0	70.55	87.7
	94.77	26.0	80.23	84.7
	73.80	25.0	60.58	82.1
	73.46	30.0	58.91	80.2
	65.50	28.0		
	69.10	34.0		
	106.82	24.0		
	120.93	27.0		
	33.41	26.0		
	117.65	27.0		
	47.26	31.0		
	155.82	36.0		
	45.78	33.0		
	91.64	25.0		
	216.27	26.7		
	124.44	27.1		
***Hypostomus hondae (D)***	93.30	27.2	77.96	83.6
	64.61	35.4	51.03	79.0
	56.45	29.3	46.50	82.4
	11.65	25.3	9.59	82.3
	77.30	24.1	64.45	83.4
	30.12	17.5	22.97	76.3
	25.00	16.7	19.11	76.4
	27.05	20.5	21.44	79.3
	58.74	27.6	43.32	73.8
	17.36	19.4	13.40	77.2
	16.35	16.2		
	35.51	22.8		
	10.51	15.7		
	5.48	14.5		
	7.47	16.7		
	6.19	18.6		
	6.85	15.8		
	26.96	28.0		
	31.74	27.0		
	22.04	25.0		
	163.18	50.0		
***Cyphocharax magdalenae (D)***	224.80	18.0	200.12	89.0
	113.40	10.2	93.54	82.5
	35.10	11.3	28.40	80.9
	129.50	15.4	110.60	85.4
	12.90	9.8		
	40.01	11.7		
	38.51	11.3		
	41.67	13.8

**Table 5:** Total mercury concentrations (THg), total length, methylmercury concentrations (MeHg) and percentage of methylmercury in fish captured in the Ciénaga de Unguía & Tumaradó (CUT) zone, Atrato River Basin, Colombia. P: piscivore, C: carnivore, OC: omnivore with a tendency to carnivore, O: omnivore, D: detritivore. The Ciénaga de Unguía is located in the municipality of Unguía in the northern zone of the Chocó Department (8 °01′11′′ N 77 °02′28′′ W). The Ciénaga de Tumaradó is located in the municipality of Turbo in the northwestern zone of the Antioquia Department (7 °46′12′′ N 77 °02′42′′ W).

Species	THg (µg kg^−1^)	Total length (cm)	MeHg (µg kg^−1^)	% MeHg
***Ageneiosus pardalis (P)***	547.95	36.9	455.04	83.0
	88.13	34.1	70.52	80.0
	67.62	27.0	55.12	81.5
***Hoplias malabaricus (P)***	235.95	33.9	196.81	83.4
	146.05	34.6	122.72	84.0
	150.83	32.9	125.91	83.5
	169.28	27.8	153.28	90.5
	208.34	43.1		
	653.31	38.0		
	80.65	34.0		
***Trachelyopterus fisheri (P)***	183.69	21.2	154.73	84.2
	182.45	21.0	165.55	90.7
	91.22	17.6	76.21	83.5
***Ctenolucius beani (C)***	200.67	21.0	186.03	92.7
	225.26	21.4	205.37	91.2
	184.56	22.0	152.02	82.4
	200.77	18.5	192.06	95.7
***Caquetaia umbrifera (C)***	113.50	25.0	92.18	81.2
	68.02	24.5	50.65	74.5
***Sternopygus macrurus (C)***	173.03	67.0	153.11	88.5
	115.32	58.0	100.18	86.9
	177.03	69.0		
***Caquetaia kraussii (OC)***	184.30	24.0	146.66	79.6
	183.70	22.8	148.82	81.0
	87.90	23.2	80.75	91.9
	70.24	21.0		
	167.40	19.5		
	64.82	19.5		
***Rhamdia quelen (OC)***	124.49	27.7	91.11	73.2
	40.00	25.8	31.97	79.9
	89.58	23.7	66.98	74.8
	55.83	24.0	46.76	83.8
	49.05	29.0		
	38.24	26.5		
	43.00	24.0		
***Astyanax fasciatus (OC)***	56.13	16.1	49.10	87.5
	136.35	14.1	123.40	90.5
	59.44	15.0	48.76	82.0
***Pimelodus punctatus (OC)***	43.82	26.5	32.81	74.9
	48.72	23.0	40.16	82.4
***Leporinus muyscorum (O)***	46.40	26.1	40.75	87.8
	44.52	33.5	36.47	81.9
	26.73	33.0	20.80	77.8
***Prochilodus magdalenae (D)***	106.97	28.9	96.39	90.1
	52.72	28.9	39.42	74.8
	90.79	31.4	84.40	93.0
	67.04	30.3	52.86	78.8
	29.30	26.9	23.13	79.0
	64.87	26.9	54.01	83.3
	125.16	30.8	101.35	81.0
	46.69	30.9	38.40	82.2
	96.56	29.6	83.68	86.7
	81.55	32.0	58.59	71.8
	56.75	28.2	42.07	74.1
	68.83	32.5	60.90	88.5
	64.33	31.7	53.69	83.5
	96.50	30.3	81.23	84.2
	50.11	28.5	43.64	87.1
	89.99	29.2	70.31	78.1
	82.41	29.4	72.83	88.4
	93.27	27.8	72.59	77.8
	77.28	30.3	61.05	79.0
	104.34	31.7	85.11	81.6
	41.78	29.8	33.79	80.9
	83.25	29.9	69.99	84.1
	308.13	28.4		
	63.46	25.0		
	79.77	28.0		
	35.02	29.9		
	72.90	32.1		
	138.24	28.8		
***Hypostomus hondae (D)***	25.61	23.8	19.58	76.4
	67.73	40.5	54.94	81.1
	53.67	41.0	39.19	73.0
	52.82	38.4	47.20	89.4
	76.62	39.0		
	53.33	25.1		
	55.85	36.0		
***Cyphocharax magdalenae (D)***	21.20	18.5	16.12	76.0
	27.57	18.8	22.73	82.4
	28.59	19.0	21.66	75.8
	32.55	20.5	29.04	89.2

This data set was useful to describe the Spearman's correlation coefficients between THg and MeHg concentrations and the total length of the fish, with which the bioaccumulation of these pollutants could be established in fish species of each of the studied areas. The data set also served to shown the median concentration of THg and MeHg in fish tissues by species in all the areas studied, and how many analyzed samples surpassed the warning levels for populations at risk and the maximum limits recommended for human consumption of fish established by WHO [2,3]. Finally, in data set, it is shown that MeHg was the predominant form of mercury in the fish of the Atrato River Basin.

## 2 Experimental Design, Materials and Methods

### 2.1 Sampling collection

The fish samples were obtained in five areas of the Atrato River Basin, between April and December 2019, subsequently classified using specialized keys [4]. A total of 842 samples were obtained, the length and weight of the fish were measured and a portion of dorsal muscle of 20–50 grams (g) was taken. The samples obtained were stored in polyethylene bags and refrigerated until the measurement of THg and MeHg concentrations.

### 2.2 Analysis of mercury species

To determine THg concentrations, fish samples were lyophilized for several days to dryness, then 0.02 g of sample was taken and introduced into a direct mercury analyzer (DMA-80 TRICELL, Milestone Inc., Italy), according the EPA 7473 method [5]. A certified dogfish muscle standard DORM-2 (4.46 ± 0.25 µg g^−1^) from NRCC was used for process quality control. The recovery percentage of THg (99.6 ± 0.2%, *n* = 3), the detection and quantification limit were calculated based on the methodology proposed by Marrugo-Negrete et al., [6].

For the measurements of MeHg concentrations, 0.2–0.3 g of fresh fish were used, the samples were subjected to digestion in a 50 mL centrifuge tube, with 10 mL of hydrobromic acid by manual shaking. To this solution 20 mL of toluene was added, then the mixture was stirred vigorously for 2 min (min). Then, the resulting mixture was centrifuged for 10 min at 3000 rpm, subsequently 15 mL of the upper organic phase were extracted, which were added in a 50 mL tubes containing 6.0 mL of 1% L-cysteine solution. Finally, a 100 µL aliquot of the aqueous phase was taken and injected into a direct mercury analyzer [7].

## Ethics Statement

The authors declare that they have read and follow the ethical requirements for publication in Data in Brief.

## CRediT Author Statement

**Carlos Salazar-Camacho:** Writing- Reviewing and Editing. **Manuel Salas-Moreno:** Performed the experiments, and data/evidence collection. Wrote the initial draft. **Roberth Paternina-Uribe**: Supervision, visualization of the Investigation. **José Marrugo-Negrete:** Developed and designed the methodology for investigation, provided reagents, materials, instrumentation and computing resources for direct mercury analyzer, critical review, commentary and revision for the pre-publication stage. **Sergi Díez:** Supervision, revision for the pre-publication stage**.**

## Declaration of Competing Interest

The authors declare that they have no known competing financial interests or personal relationships which have or could be perceived to have influenced the work reported in this article.

## References

[1] C. Salazar-Camacho, M. Salas-Moreno, R. Paternina-Uribe, J. Marrugo-Negrete, S. Diez, Mercury species in fish from a tropical river highly impacted by gold mining at the Colombian Pacific region, Chemosphere. 10.1016/j.chemosphere.2020.128478 33065322

[2] World Health Organization (1990). Methylmercury in Environmental Health Criteria 101. https://apps.who.int/iris/bitstream/handle/10665/38082/9241571012_eng.pdf.

[3] World Health Organization, “Guidance for Identifying Populations at Risk from Mercury Exposure”. Issued by: UNEP DTIE Chemicals Branch and WHO Department of Food Safety, Zoonoses and Foodborne Diseases. Geneva, Switzerland, 2008. https://wedocs.unep.org/bitstream/handle/20.500.11822/11786/IdentifyingPopnatRiskExposuretoMercury_2008Web.pdf?sequence=1&isAllowed=y [Accessed: 16 September 2020].

[4] FishBase., 2020. [WWW Document]. URL. FishBase http://www.fishbase.org/search.php. [Accessed: 16 September 2020].

[5] EPA., 1998. United State Environmental Protection Agency. Method 7473 (SW-846). Mercury in solids and solutions by thermal decomposition, amalgamation, and Atomic Absortion Spectrophotometry". Washington DC. https://www.epa.gov/sites/production/files/2015-07/documents/epa-7473.pdf [Accessed: 16 September 2020].

[6] Marrugo-Negrete J., Vargas S., Ruiz J., Marrugo-Madrid S., Bravo A., Díez S. (2020). Human health risk of methylmercury from fish consumption at the largest floodplain in Colombia. Environ. Res..

[7] F. Cordeiro, S. Gonçalves, J. Caldéron, P. Robouch, H. Emteborg, P. Coneel, M. Tumba-Tshilumba, B. Kortsen, B. de la Calle, 2013. IMEP-115: determination of Methylmercury in Seafood, Issue February 2013. 10.2787/76278. 24830172

